# Otosclerosis under microCT: New insights into the disease and its anatomy

**DOI:** 10.3389/fradi.2022.965474

**Published:** 2022-08-05

**Authors:** Gabriela O'Toole Bom Braga, Robert Zboray, Annapaola Parrilli, Milica Bulatović, Marco Domenico Caversaccio, Franca Wagner

**Affiliations:** ^1^ARTORG Center for Biomedical Engineering Research, University of Bern, Bern, Switzerland; ^2^Center for X-ray Analytics, Swiss Federal Laboratories for Materials Science and Technology (Empa), Dübendorf, Switzerland; ^3^Department of Otorhinolaryngology, Head and Neck Surgery, Inselspital, University Hospital Bern, Bern, Switzerland; ^4^Department of Diagnostic and Interventional Neuroradiology, Inselspital, University Hospital Bern, Bern, Switzerland

**Keywords:** otosclerosis, microCT, 3D anatomical study, cochlea, tonotopic mapping, porosity analysis

## Abstract

**Purpose:**

Otospongiotic plaques can be seen on conventional computed tomography (CT) as focal lesions around the cochlea. However, the resolution remains insufficient to enable evaluation of intracochlear damage. MicroCT technology provides resolution at the single micron level, offering an exceptional amplified view of the otosclerotic cochlea. In this study, a non-decalcified otosclerotic cochlea was analyzed and reconstructed in three dimensions for the first time, using microCT technology. The pre-clinical relevance of this study is the demonstration of extensive pro-inflammatory buildup inside the cochlea which cannot be seen with conventional cone-beam CT (CBCT) investigation.

**Materials and Methods:**

A radiological and a three-dimensional (3D) anatomical study of an otosclerotic cochlea using microCT technology is presented here for the first time. 3D-segmentation of the human cochlea was performed, providing an unprecedented view of the diseased area without the need for decalcification, sectioning, or staining.

**Results:**

Using microCT at single micron resolution and geometric reconstructions, it was possible to visualize the disease's effects. These included intensive tissue remodeling and highly vascularized areas with dilated capillaries around the spongiotic foci seen on the pericochlear bone. The cochlea's architecture as a morphological correlate of the otosclerosis was also seen. With a sagittal cut of the 3D mesh, it was possible to visualize intense ossification of the cochlear apex, as well as the internal auditory canal, the modiolus, the spiral ligament, and a large cochleolith over the osseous spiral lamina. In addition, the oval and round windows showed intense fibrotic tissue formation and spongiotic bone with increased vascularization. Given the recently described importance of the osseous spiral lamina in hearing mechanics and that, clinically, one of the signs of otosclerosis is the Carhart notch observed on the audiogram, a tonotopic map using the osseous spiral lamina as region of interest is presented. An additional quantitative study of the porosity and width of the osseous spiral lamina is reported.

**Conclusion:**

In this study, structural anatomical alterations of the otosclerotic cochlea were visualized in 3D for the first time. MicroCT suggested that even though the disease may not appear to be advanced in standard clinical CT scans, intense tissue remodeling is already ongoing inside the cochlea. That knowledge will have a great impact on further treatment of patients presenting with sensorineural hearing loss.

## Introduction

Otosclerosis was first reported by Valsalva in 1735 and described as an otospongiotic lesion in 1912 by Cureoglu et al. (1). Guild (2) and Schuknecht (3) characterized it as a disorder that affects the bony labyrinth and the stapes causing conductive and sensorineural hearing loss (SNHL) (1, 4, 5). Whereas, otospongiosis is described as replacement of the endochondral layer of the otic capsule by a markedly more vascularized spongy bone, otosclerosis occurs when these lesions become calcified and sclerotic.

Clinically, otospongiosis presents as lesions (cavitary plaques) on the stapedial annular ligament causing stapes fixation, which leads to conductive hearing loss, especially at lower frequencies. With the progression of the disease, the cochlea becomes involved resulting in mixed hearing loss and SNHL. The characteristic behavior of hearing loss in patients with otospongiosis helps in making an early and accurate clinical diagnosis. Additionally to the physical exam, basic hearing tests identify specific signs of the disease, such as: hearing loss that is greater for low frequencies in air conduction, Carhart notch (a classic sign of the disease that affects the 2,000 Hz frequency in bone conduction), Weber test (stimulus perception in the ear with conductive hearing loss), shallow type A audiogram, absence of stapedial acoustic reflex, and absence of otoacoustic emissions (6). A cone-beam computed tomography (CBCT) scan or a conventional CT scan complements the clinical diagnosis and provides additional anatomical information (i.e., on facial nerve canal dehiscence), which assists in surgical planning.

Otospongiotic cavitary plaques can be seen on CT as focal lesions, known as spongiotic foci, around the cochlea. The fissula ante fenestram (FAF) and the inferior walls of the internal auditory canal (IAC) are the most commonly affected areas. The FAF is a small cleft, composed of connective tissue and located anterior to the oval window (OW) (7). This tissue will later calcify and cause the stapes fixation. The anticipated hearing loss is treated with a conventional hearing aid or surgery, where a stapes prosthesis (titanium or Teflon) is used to replace the affected bone. Although the diagnosis is mainly clinical, evaluation using pre-operative imaging is pertinent. Current CT resolution can reveal very subtle bony changes, which even allow detailed anatomical evaluation using grading systems (7). Nonetheless, the resolution remains insufficient to enable definitive intracochlear damage evaluation. MicroCT technology provides resolution at the single micron level, offering an exceptional amplified view of the otosclerotic cochlea, which allows a precise tridimensional reconstruction.

A radiological and a three-dimensional (3D) anatomical study of an otosclerotic cochlea, made using microCT technology, is presented here for the first time. 3D segmentation of the human cochlea was performed, providing an unprecedented view of the diseased area without the need for decalcification, sectioning, or staining. Using microCT at single micron resolution, it is possible to visualize the disease's effects on the surrounding bone as well as the cochlea's architecture using geometric reconstruction. Given the recently described importance of the osseous spiral lamina (OSL) in hearing mechanics (8, 9) and that, clinically, one of the signs of otosclerosis is the Carhart notch observed on the audiogram, a tonotopic map using the OSL as region of interest is also presented. An additional quantitative study of the porosity and width of the OSL is also reported.

## Materials and methods

The authorization of the local institutional review board (KEK Bern, Switzerland, Project-ID 2018-00770) was obtained. A cadaveric sample of a human head (formalin flushed) with bilateral signs of stapedectomies, characterized by the presence of a stapes prosthesis in the middle ear, was used for this study (*n* = 1). The left side was randomly chosen for this experiment. The diagnosis of otosclerosis/otospongiosis was confirmed by the existence of a stapes prosthesis in the middle ear. It was also confirmed radiologically, after the evaluation of the images, by an experienced neuroradiologist specialized in head and neck imaging and an experienced otologic surgeon. In addition to the imaging analysis performed, the serologic tests and the patient's medical history, which was provided with the specimen, excluded other diseases, including the differential diagnosis of osteogenesis imperfecta, Paget's disease or Lobstein disease. However, it did not exclude measles whose role in the pathogenesis of the disease remains unclear (10).

### Cochlea removal

**Figure 1:**
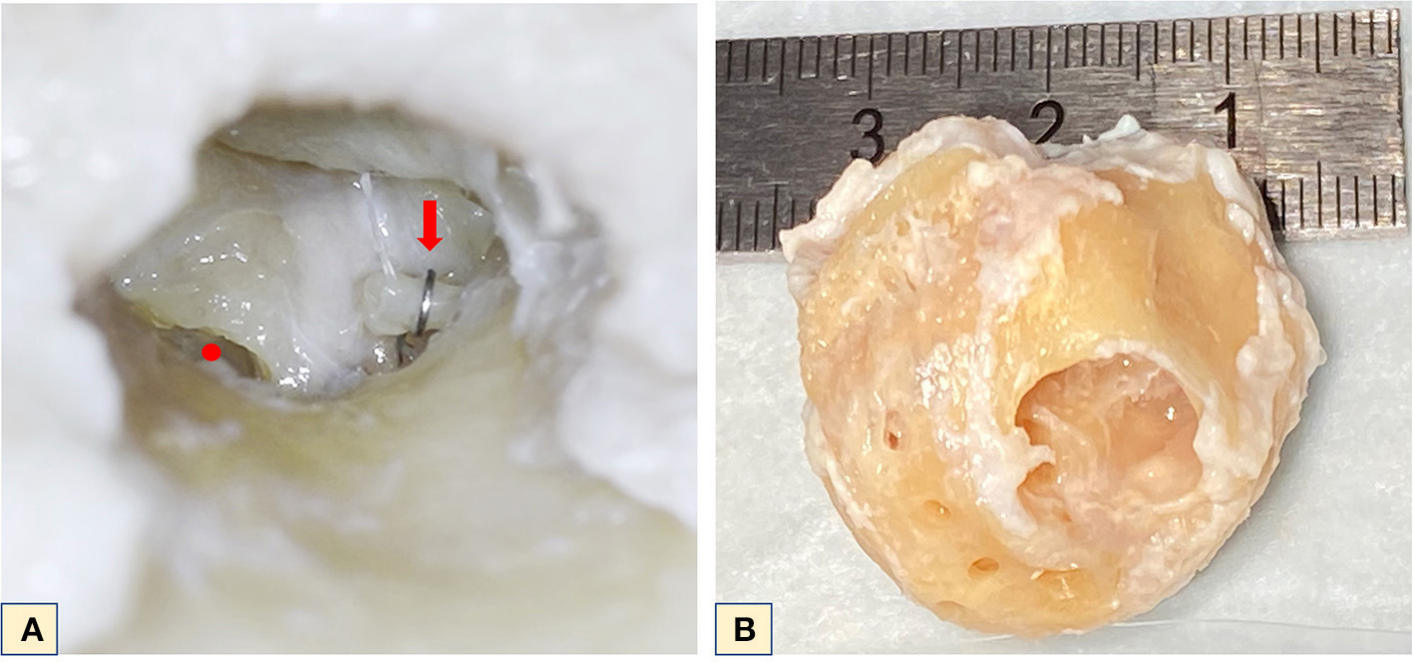
**(A)** Microscopic middle ear view of the left cochlea. Red dot–round window; red arrow–stapes prosthesis positioned on the long process of the incus. **(B)** Final cochlea size after refinement for microCT.

Owing to limitations on the detector size and to avoid excessive parasitic absorption by the surrounding bone structures, the cochlea's surrounding otic capsule had to be removed. Prior to cochlea refinement, the temporal bone was scanned using CBCT (voxel size 0.15 × 0.15 × 0.2 mm^3^, XCAT XL, Xoran, MI, USA). The dissection started with middle ear inspection, which revealed an artificial titanium stapes prosthesis (Figure 1A) confirming that the patient had a clinical diagnosis of otospongiosis. A post-auricular incision was made, and the mastoid bone was exposed. Later, a mastoidectomy and subsequent labyrinthectomy, as described by Glasscock (11), was performed. These procedures remove the bone surrounding the cochlea, which includes the semicircular canals, part of the vestibule and middle ear structures, leaving the cochlea exposed. The stapes prosthesis was kept in place for anatomical reference. The VII and VIII cranial nerves of the IAC were detached from the skull base and the cochlea was removed from its encasement. At this point, the cochlea went through a dissection refinement process until it was no more than 1.5 cm in diameter (Figure 1B).

### MicroCT

MicroCT analysis was carried out using an EasyTom XL Ultra 230–160 micro/nanoCT scanner (RX Solutions, Chayanod, France). The scanner features a Hamamatsu nano-focus, transmission X-ray tube with a 1-μm thick tungsten target on a diamond window. The tube was operated with a LaB6 cathode. The scans were performed using a Varian PaxScan 2520DX detector (flat panel with amorphous silicon and a CsI conversion screen; 1,920 × 1,536-pixel matrix; pixel pitch of 127 mm; 16 bits of dynamic range). The tube was operated at 70 kV and a current of 60 μA. To allow a more detailed reconstruction, the specimen was scanned using a protocol specifically designed to visualize bony structures inside the cochlea (such as the OSL). Subsequently, two scans of the full cochlea with different voxel sizes were performed: One with 6.63 μm nominal resolution (voxel size) that provides detailed visualization of the round and oval windows, and the other with 4.46 μm nominal resolution that provides a detailed reconstruction of the OSL, but no full view of the windows. To visualize the basal and middle turns and apex, additional zoom scans at voxel sizes of 2.35, 2.41 and 2.48 μm, respectively, were performed using the same tube setting as for full cochlea scans.

### 3D-segmentation and frequency map formulation

For the 3D reconstructions of the cochlear bone, capillary system, OSL, and intracochlear calcifications, the medical image analysis software Amira (Thermo Fisher Scientific, Waltham, Massachusetts, USA), was used. For the full cochlea, and the basal and midturns, threshold adjustment was used to create the 3D images. The bone was segmented, and the selected voxels were added to a label; any noise was removed using the brush tool. This label was later multiplied by the original. Tiff image to isolate the labeled anatomy and, using the volume rendering tool, the 3D mesh image was created. At the apex a different approach was necessary owing to the predominance of spongiotic bone and fibrotic tissue. Using threshold adjustment, the soft tissue was segmented, and the selected voxels were added. On another label, the bone was segmented, and the selected voxels were subtracted. Those labels were multiplied by the image and, using the volume rendering tool, the mesh image was created. In this case the noise was not removed, as it corresponded to the soft tissue seen on the raw image filling up the compartment. The same color map was applied and adjusted for each image.

The segmented images were then further analyzed in Mimics (Materialize NV, Leuven, Belgium). On the mesh object, spiral ganglion (SG) and the inner and outer walls of the OSL were contoured using the spline function. The middle axis was set by considering the helicotrema, the center point of the cochlea, and used as a reference axis. Following contouring, the measurements were conducted on reconstructed splines in a custom script (Python 3.9.0). The OSL width was calculated from apex to base as the distance between the inner (modiolus) and the outer wall (the lateral end of the OSL) along the reference axis. Finally, the OSL was divided into tonotopic frequency partitions using a similar approach to that described by Li et al. (12). In their study, the SG was partitioned into tonotopic partitions by dendrite tracing from the basilar membrane partitions (13). Similarly, in our study, Li's SG function was used to determine the octave band partitions on the SG, which were then traced to the borders of the bony OSL by following the ridges corresponding to the dendrites. Within each partition, the mean width and the standard deviation (SD) were calculated.

To estimate the porosity of the individual plates, tympanic and vestibular, at different segments of the OSL (i.e., the basal turn, the midturn, and the apex), the plates were reconstructed in 3D and exported as STL models. The volumetric porosity level of each plate segment was calculated as the ratio of the void volume over the filled volume:


$$\begin{array}{l} p=\frac{V_{f}-V_{o}}{V_{f}}\cdot 100\% \end{array}$$

where, *V*_*o*_ denotes the original (porous) volume and *V*_*f*_ its filled (non-porous) counterpart. The filling of the pores was achieved using a 3D graphics software package (Blender 2.90) by shrink-wrapping a solid bounding volume to the original parts. In this way, the pores were filled by decimating the bounding volume to wrap the plates, resulting in an approximate non-porous adaptation of the original models.

## Results

We were able to demonstrate that it is possible to visualize structural anatomical alterations using microCT technology, without the need for the decalcification, staining or sectioning usually used in histological preparations. We also used the microCT images to create 3D reconstructions of the anatomy that revealed a new perspective on how the disease affects the cochlea. Additionally, a tonotopic map was built showing the frequencies affected by the disease. The OSL width was measured as well as its porosity.

### Imaging analysis

Using the medical image analysis software Amira (Thermo Fisher Scientific, Waltham, Massachusetts, USA) the CBCT and microCT images were individually and independently evaluated by both the neuroradiologist and the otologic surgeon for signs of otosclerotic disease.

**Figure 2:**
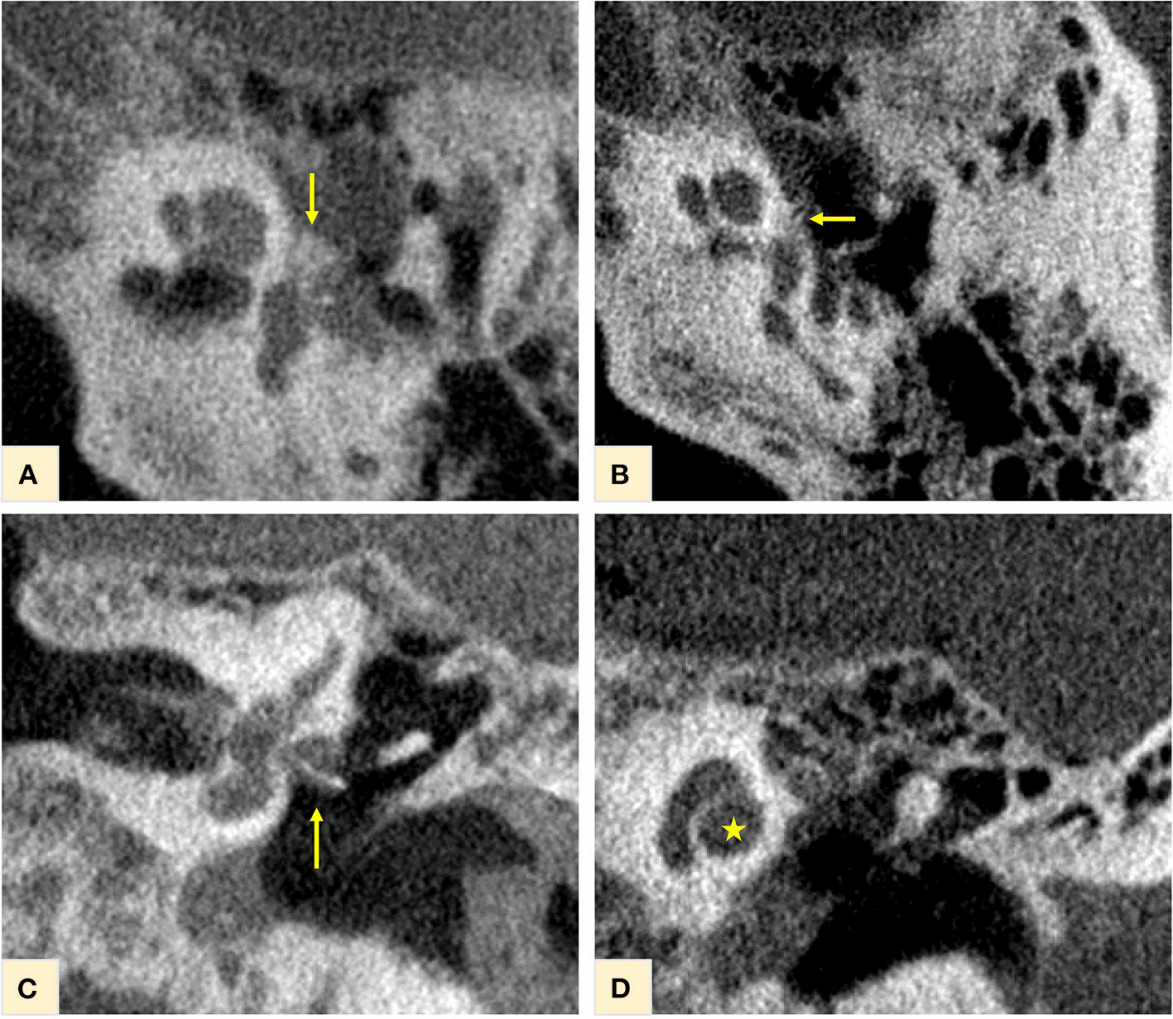
CBCT of the temporal bone. Otospongiotic plaques in *loco typico* confirming the disease diagnosis, were clearly recognizable at the fissula ante fenestrum, with involvement of the oval window and the “otosclerotic angle” [yellow arrows in **(A,B)**]. The titanium stapes prosthesis *in situ* for disease treatment is shown on **(C)** (yellow arrow). At this initial stage of the disease, no other otospongiotic foci were seen and the cochlea appeared with a normal contour on **(D)** (yellow star).

First, the CBCT scan was analyzed. Classic otospongiotic plaques on the typical loci, such as FAF and involvement of the OW at the “otosclerotic angle,” were clearly recognizable and confirmed the diagnosis. The otosclerotic angle is the region between the anterior portion of the stapes footplate, the *processus cochleariformis* and the adjacent bulge of the *promontorium* (Figures 2A,B). The titanium stapes prosthesis is seen *in situ* on Figure 2C. At this initial stage of the disease, no otosclerotic remodeling foci at other locations of the bony labyrinth, including the area of the IAC, the carotid canal, the vestibule, or the semicircular canals, were evident. In particular, there was no involvement of the otic capsule, usually present at later stages of the disease with the classic confluent “halo” demineralization (Figure 2D).

**Figure 3:**
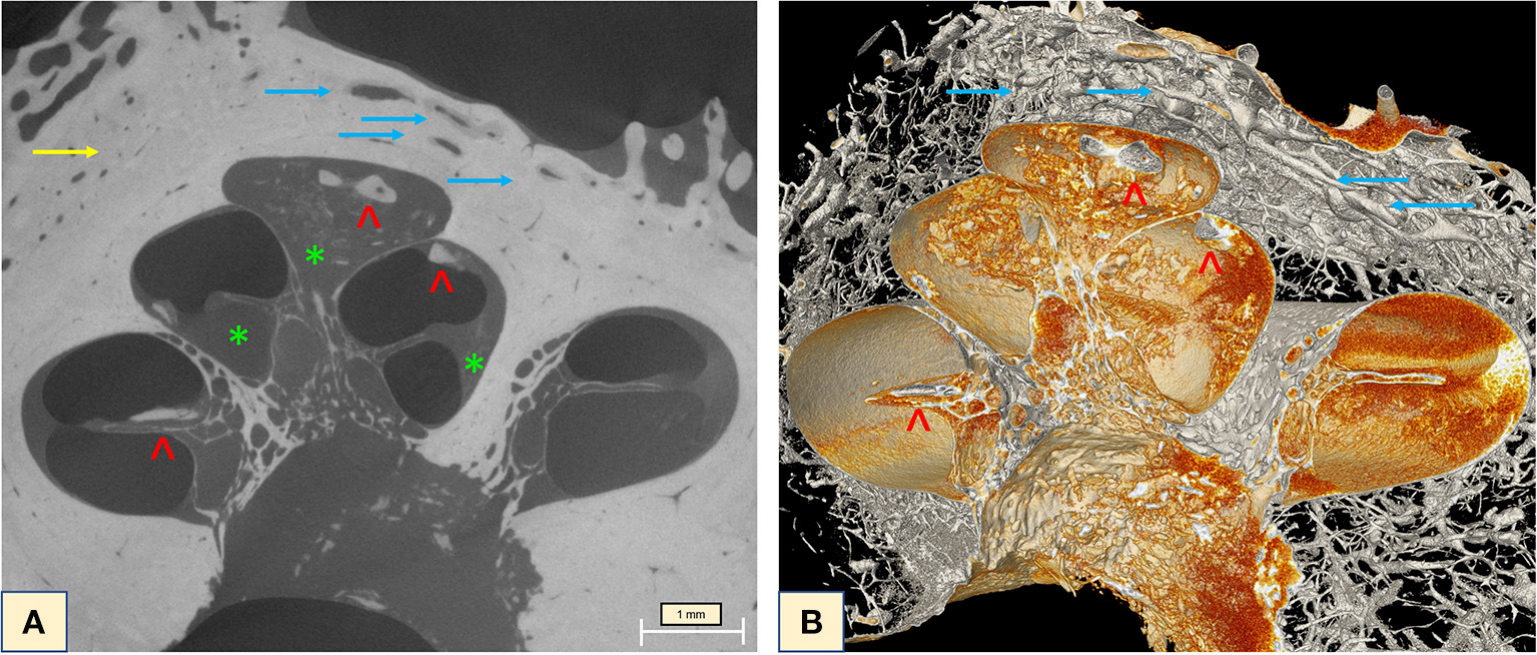
**(A)** MicroCT raw image of the full cochlea (6.63 μm) revealed intense vascularization (blue arrows) and the halo sign (yellow arrow). Extensive fibrotic tissue taking over the apex, the scala tympani of the middle turn, fibrotic tissue (green stars) and lithic formation on top of the OSL and throughout the apex (red arrowheads) were visible with excellent imaging quality. **(B)** Mesh reconstruction of the cochlea showing the lithic formations throughout the cochlea (red arrowheads) and the intense vascularization of the otospongiotic bone (blue arrows).

**Figure 4:**
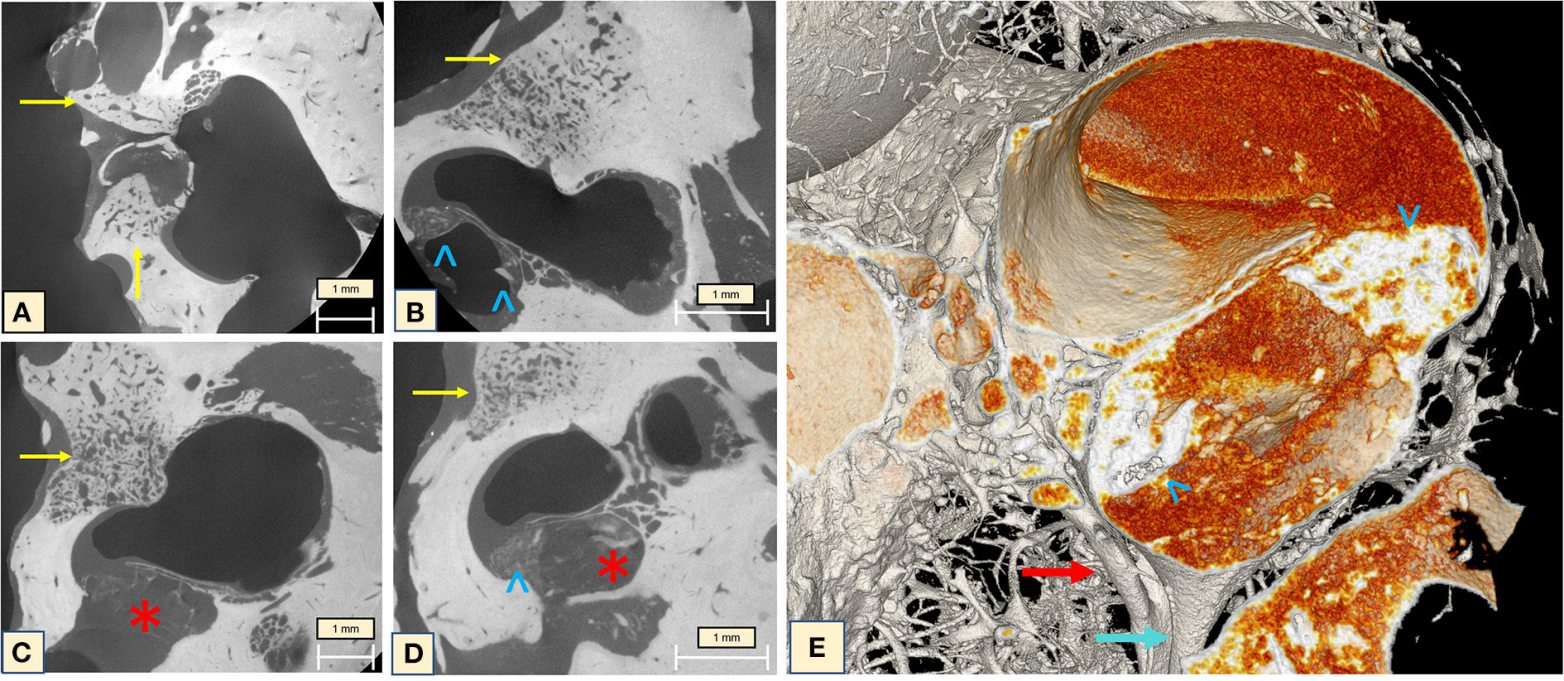
**(A)** The oval window with the fractured stapes and otospongiotic bone (yellow arrows) with multiple dilated vessels; **(B)** the oval window is surrounded by spongiotic bone (yellow arrow) and lithic formation of the CP and under the OSL (blue arrowheads); **(C,D)** otospongiotic bone (yellow arrows) surrounding the round window and fibrotic tissue taking over the scala tympani region (red star) and lithic formations (blue arrowheads). **(E)** 3D mesh reconstruction of the region immediately posterior to the round window (RW), reveals intense dense bone formation on the CP (blue arrowheads) and the cochlear aqueduct (CA) (blue arrow) and the inferior cochlear vein (ICV) next to it (red arrow). The orange image taking over the compartment corresponds to the fibrotic tissue seen on the microCT. Note how the fibrotic tissue (“red cloud”) obliterates both CA and ICV.

Investigation of the full cochlea at 6.63 μm resolution microCT showed different attenuation signals and density values on the otic capsule with increased vascularity. In addition, fibrotic plaques were seen in all cochlea turns, as well as lithic lesions, here called cochleoliths (CLs). The cochlea apex seemed to be the most affected anatomical area, with mixed fibrotic and dense calcified plaques covering the apex in full extension. The fibrotic tissue and the calcified plaques were also seen at the modiolus, IAC, spiral ligament (SL) and over the OSL. On the 3D reconstruction it was possible to visualize the real extent of the highly vascularized areas with dilated capillaries around the spongiotic areas seen on the microCT. When a sagittal cut was made on the 3D mesh, it was possible to visualize intense ossification of the cochlear apex, as well as the IAC, the modiolus, the SL, and a big cochleolith over the OSL (Figure 3). Additionally, the oval and round windows were examined with the 6.63 μm resolution and showed intense fibrotic tissue formation, spongiotic bone at the FAF seen as increased vascularization and CL over the cochlear partition (Figures 4A–E). At the round window, the CL appeared to connect to the OSL (Figures 4C,D). The cochlear partition (CP) region was thickened and had completely lost its normal appearance, when compared to other microCT or synchrotron images of the human cochlea (Figure 4B) (14, 15). The round window membrane was also thickened with CL and the stapes footplate was fractured, with debris invading the vestibule (Figure 4A). Imaging inspection showed excessive fibrotic and calcified tissue over the cochlear aqueduct and inferior cochlear vein (ICV) seen on the mesh reconstruction (Figure 4E).

**Figure 5:**
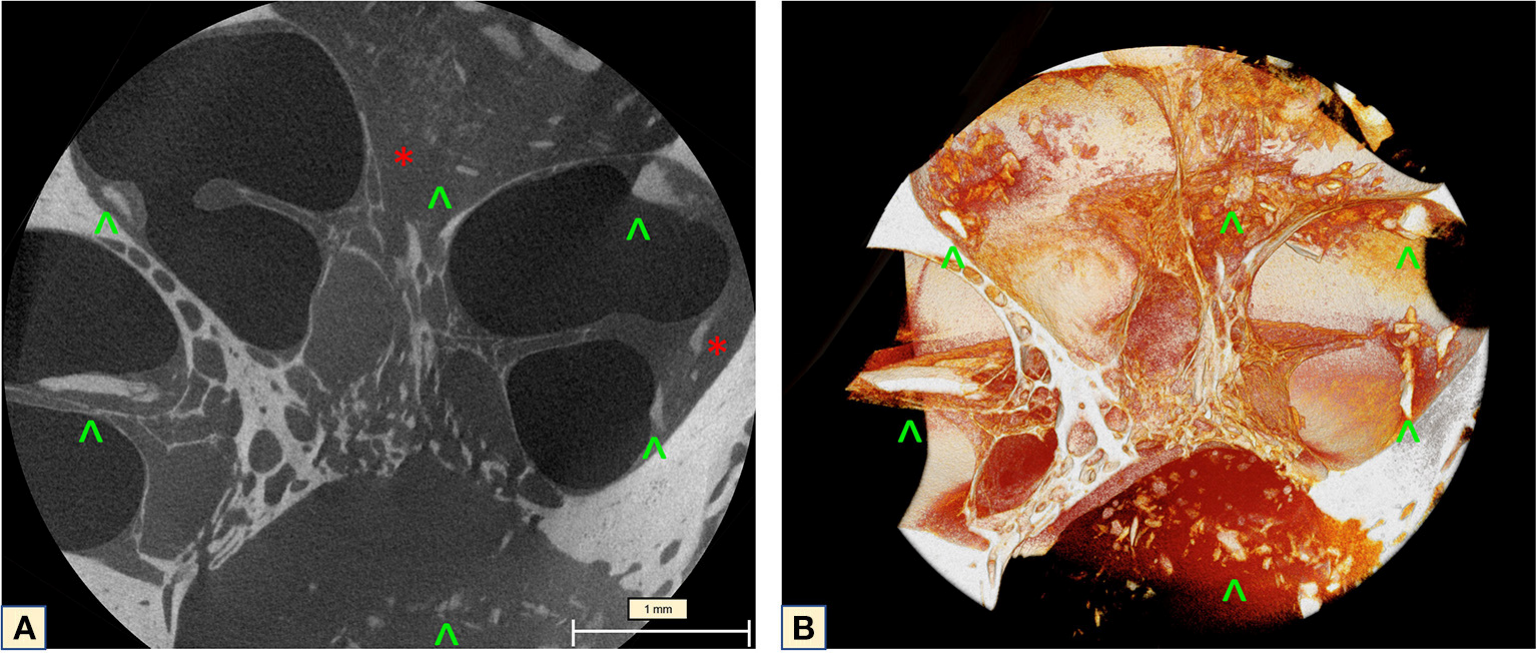
MicroCT of the cochlea midturn (2.41 μm): **(A)** Scattered dense lithic formations (green arrowheads) at the IAC, SL, over the OSL and the apex, combined with extensive fibrotic tissue formation (red stars). **(B)** Mesh reconstruction in 3D: diffuse distribution of the lithic lesions throughout the wall of the cochlea, the CP, IAC and over the OSL (green arrowheads).

MicroCT of the midturn (2.41 μm) allows an overall visualization of the ossification process inside the cochlea, focusing on the OSL and enabling concomitant SG evaluation. During imaging analysis, diffuse fibrotic tissue formation in the modiolus was observable, with ossifying plaques continuously from the cochlear apex toward the base of the cochlea. A thickening of the CP associated with high-density bone in the region of the basilar membrane and the SL was also observed. On the 3D mesh reconstructions, it was possible to visualize the CL on the cochlear wall and over the OSL. The IAC was also affected by the cochlear ossification process (Figure 5).

**Figure 6:**
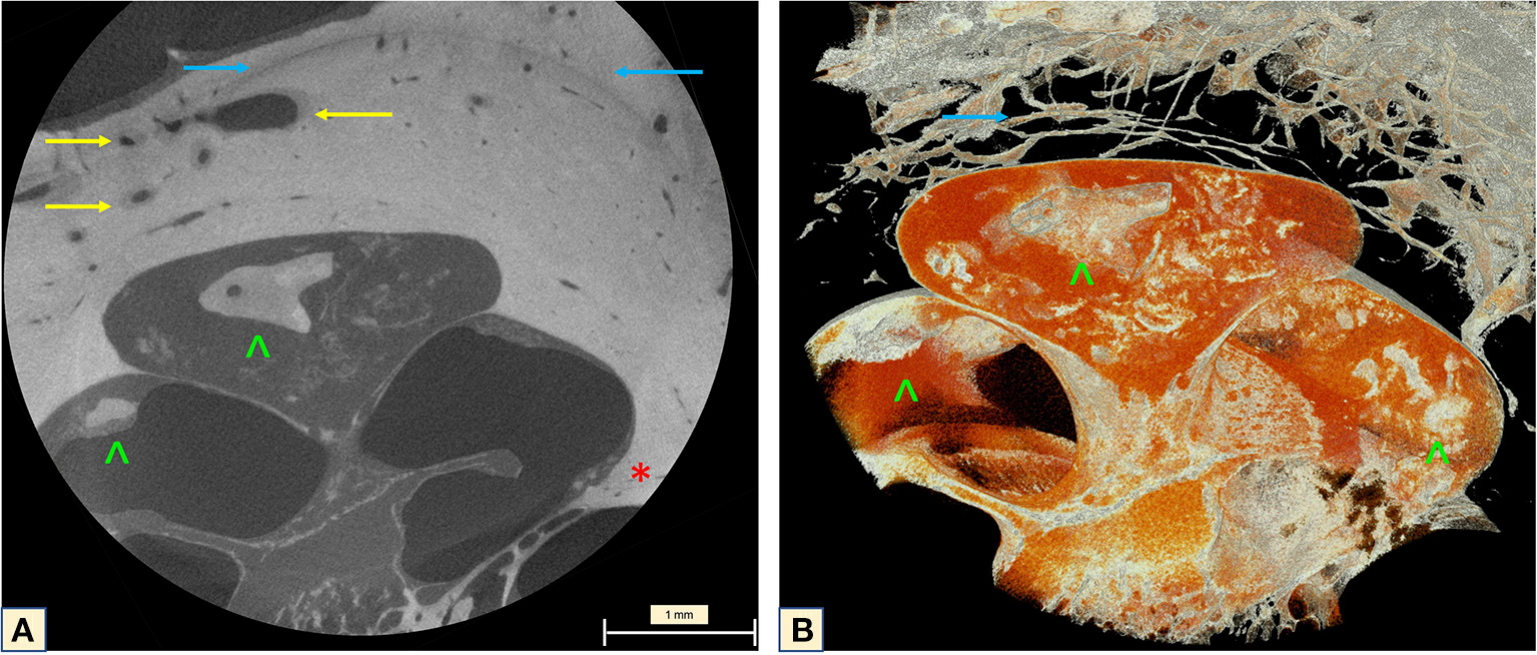
MicroCT of the cochlear apex (2.48 μm). **(A)** Neovascularization with enlarged vessels in the region of the otospongiotic bone (yellow arrow). The classic halo sign with different density of the bony matrix around the cochlea is indicated by the blue arrow. An impressive carp-head-shaped cochleolith is seen on the apex (green arrowhead) as well as intense fibrotic tissue formation. Irregularities are visible on the bony cochlear wall at the transition zone to the spongiotic bone (red star). **(B)** Mesh reconstruction of the apex demonstrating the high vascularization of the halo region (yellow arrows) and the lithic plaques (green arrowheads). The orange image taking over the apex compartment corresponds to the fibrotic tissue seen on the microCT.

The cochlear apex seemed to be the most affected area. The apex study (2.48 μm) showed that the fibrotic tissue completely filled the last spiral, covering the OSL and containing CLs. The SL and the modiolus presented the same aspect on imaging (Figure 6). Attention should be paid to the optic capsule bone. This bone presents a lithic aspect, with a change of the density surrounding the dilated capillary vessel network, and spongiotic aspect with expansive character and confluent vessels. Subtle irregularities were seen at the cochlear wall next to the spongiotic areas (Figure 6A), but when visualized with 3D reconstructions (Figure 6B) these lithic formations became clearer and were visible even on the cochlear walls. This same characteristic was noticed in other slices of the apex, demonstrating the advanced stage of the disease.

### OSL width, tonotopic and porosity analysis

**Figure 7:**
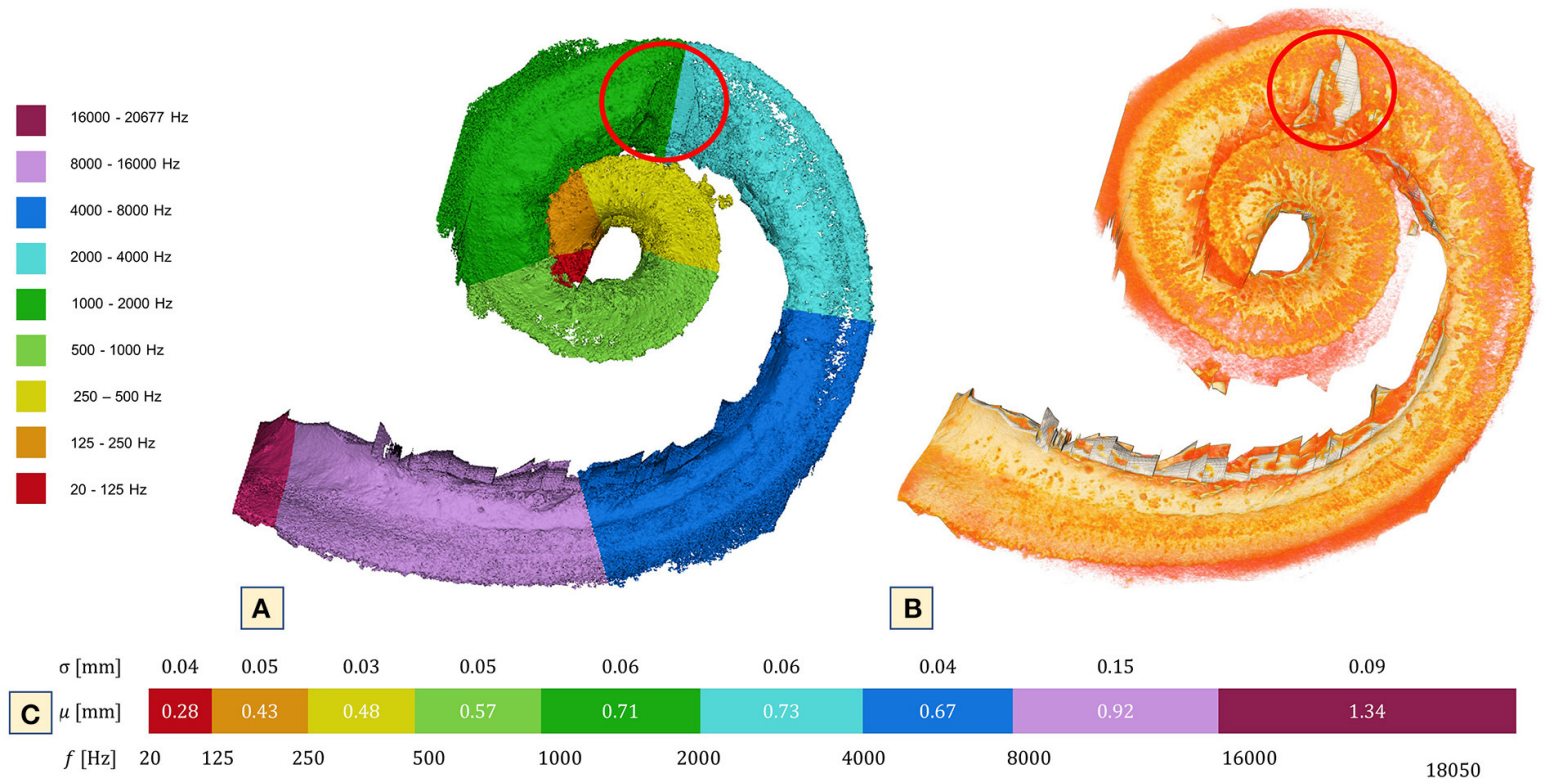
3D reconstruction of the OSL at 4.46 μm voxel size. **(A)** Tonotopic map of the cochlea using the OSL as reference and the bony formation at 2,000 Hz. **(B)** Simple segmentation of the OSL (orange) and a clear view of the lithic process over the 2,000 Hz (red circle). Note that only in that particular part of the OSL is it possible to see the bone formation. The region that corresponds to the CP was not included in this segmentation. Nonetheless, CL were present throughout its extension. **(C)** Mean and SD of cochlea width measured according to octave band partitions.

The OSL width showed the expected descending behavior. Mean width of the OSL was found to be between 1.34 and 0.28 mm with a SD of 0.09 ± 0.04. Due to the OSL's radiopaque characteristics and because it houses the nerve fibers that lead toward the SG and form the cochlear nerve, this structure was used as the reference to create the tonotopic map (Figure 7). The map (4.46 μm) indicates that the cochleolith seen over the OSL is localized in the topographic region of the 2 kHz (Figure 7A). No CLs were found in other regions of the OSL. The SL and the stria vascularis, which forms the cochlear wall (15), were covered in these CLs, which also affect the CP and the scalas, as observed during imaging analysis.

**Figure 8:**
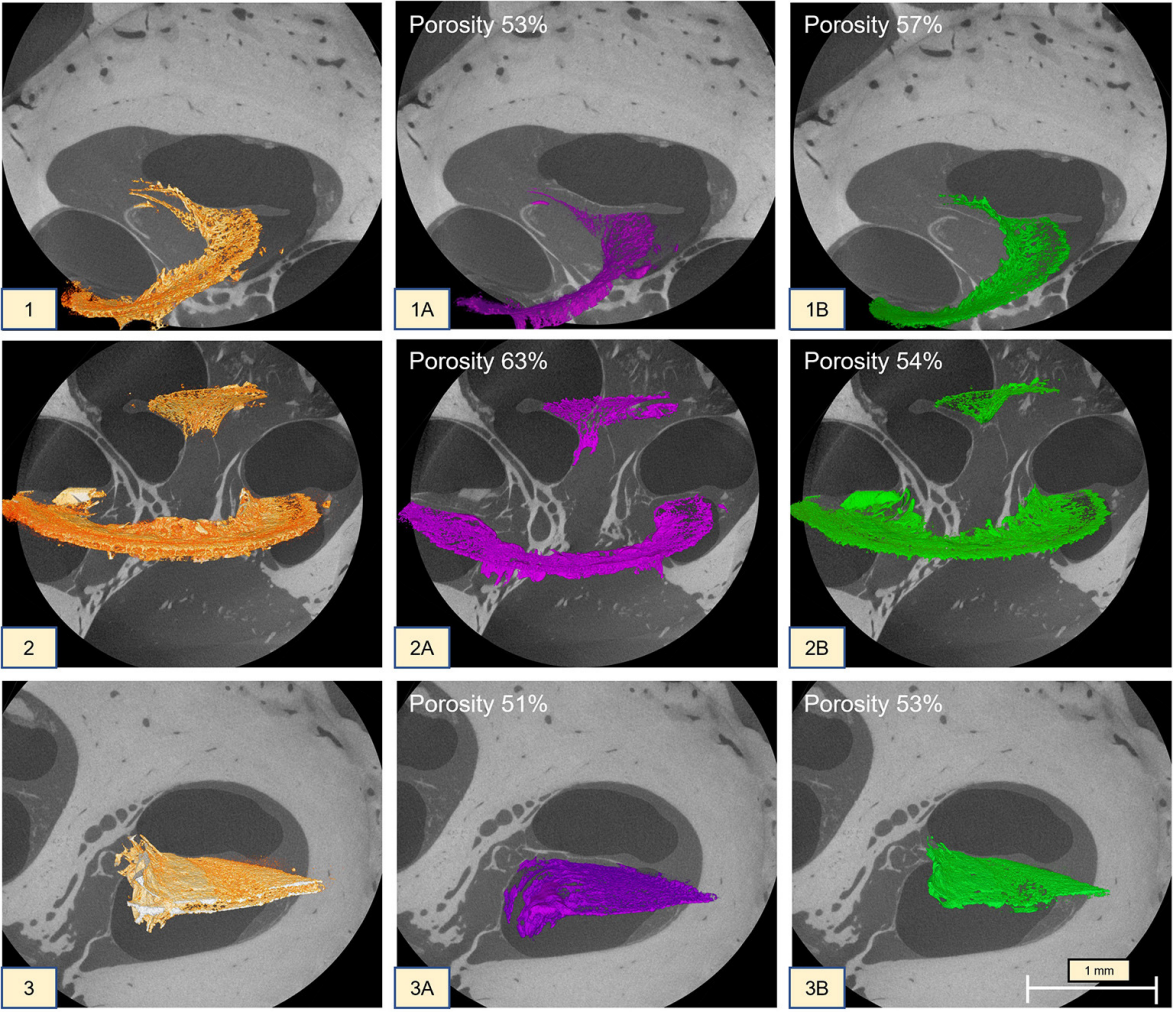
MicroCT (1 at 2.35 μm; 2 at 2.41 μm; 3 at 2.48 μm) of the cochlea turns. (1) Apex OSL; (1A) tympanic plate with 53% porosity; (1B) vestibular plate with 57% porosity; (2):midturn OSL (the CL was kept on the segmentation); (2A) tympanic plate with 63% porosity; (2B) vestibular plate with 54% porosity; (3) basal turn OSL; (3A) tympanic plate with 51% porosity; (3B) vestibular plate with 53% porosity.

Slice-by-slice inspection of the OSL plates and porosity analysis demonstrated similar porosity percentages between the basal turn and the apex, with a more porous vestibular plate (VP). In contrast, the midturn exhibited a more porous tympanic plate (TP); the results are summarized in Figure 8.

### Anatomical observations of a healthy and an otosclerotic cochlea

**Figure 9:**
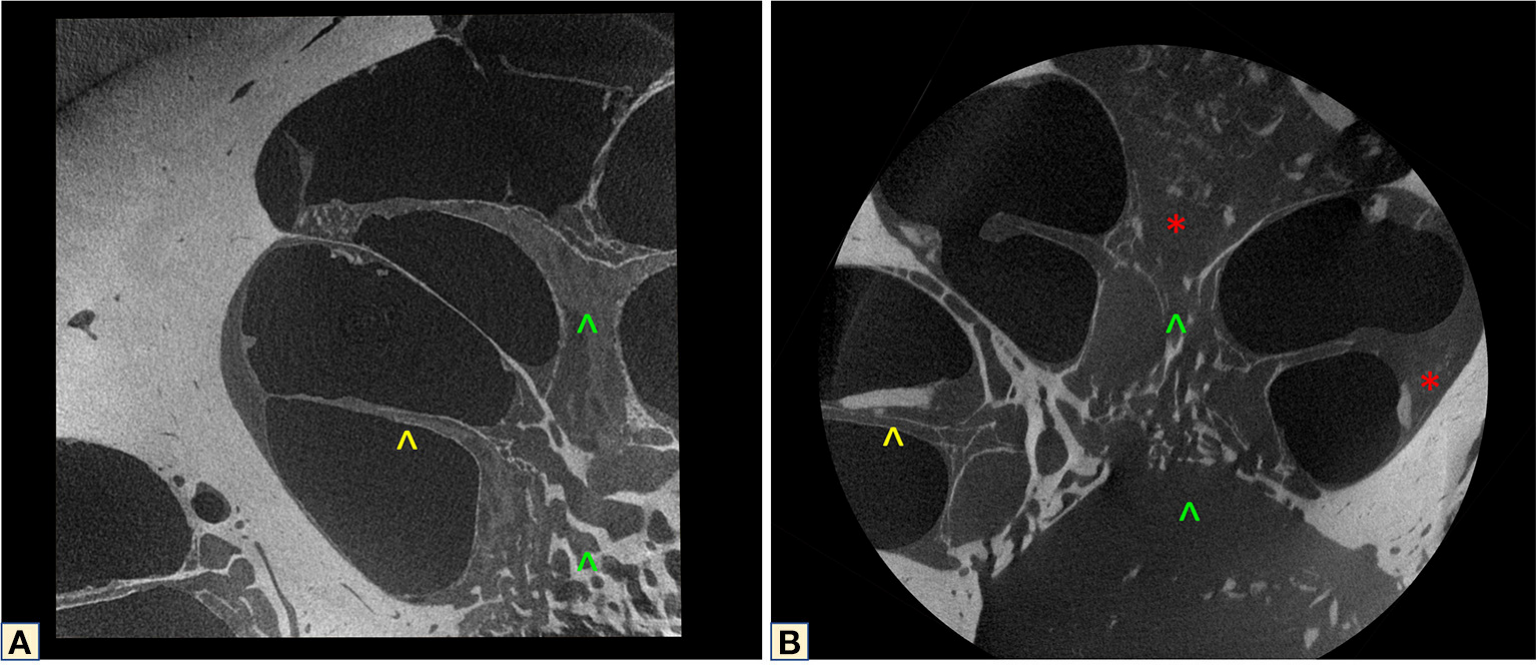
MicroCT correlation between a healthy **(A)** and an otosclerotic cochlea **(B)**. Yellow arrowhead shows the normal OSL, and the disease-affected structure. Green arrowheads demonstrate the different modiolus density of the two cochleas. The red star shows the fibrotic tissue.

A normal cochlea was scanned to allow anatomical comparison to the otosclerotic cochlea. Using 2.4 μm reconstruction of the middle turn, it was possible to observe more ossification on the modiolus, while the OSL, CP and cochlear walls had no CL. The otosclerotic cochlea, however, presented extensive fibrotic formations on the cochlear walls, CP and OSL. Cochleolith formations and the decrease in bone density on the IAC and modiolus, compared to the normal cochlea provides an unequivocal demonstration of the anatomical differences between a healthy and an otosclerotic cochlea (Figure 9).

## Discussion

This study investigated the effects of otosclerosis/otospongiosis in the cochlea using microCT. The study used single-digit-micron voxel-sized raw image data from an untouched (unstained and non-decalcified) cochlea to obtain detailed 3D reconstructions of the anatomical consequences of otospongiosis, while avoiding the artifacts introduced by histopathology techniques. Although current CT can provide sufficient information regarding the effects of the disease on the stapes, cochlear ossification and architectural changes are not seen. However, the bony dyscrasia expressed by vascular circumscribed foci of otospongiosis remodeling the bony labyrinth, is usually well seen on CBCT and conventional CT (16). In the same way fenestral otosclerosis, where the FAF and the cartilaginous remnants of the endochondral bone near the OW–known as the “*locus minoris resistentiae*” due to the presence of highly vascularized otospongiotic foci–is also observed (17). Nonetheless, unseen intense inflammatory changes are already happening inside the cochlea, potentially contributing to the progression of the disease.

MicroCT inspection identified an unexpected extensive inflammatory bone response, which translated as bone remodeling through increased vascularity. Even though these alterations are not visible in CBCT, on single-digit-micron voxel-sized images it is possible to see the confluent halo demineralization, especially on the cochlear apex and also surrounding the rest of the cochlea, including the IAC. Intensified vascularization, CLs and different density of the bony matrix is also found around and inside the IAC. Rüedi (18), Rüedi and Spoendlin (19), and Elonka and Applebaum (20) suggested that vascular shunts between the otosclerotic foci and the capillaries in the modiolus, venous congestion and stasis in the cochlear veins or thrombotic vessels of the IAC, could be a cause of SNHL in patients with otosclerosis. Although no stasis or thrombotic vessels could be seen in our images due to devascularization of the cadaveric specimen, the enlarged and confluent remodeling of vascular spaces observed in this dataset is indicative of a pathologic blood vessel proliferation (19). This vascular behavior was described by Rüedi (18) and Rüedi and Spoendlin (19) as shunts between the vessels of the active otosclerotic foci and the spiral veins. Additionally, fibrotic tissue and calcifications were visualized on top of the ICV and the cochlear aqueduct, possibly offering support for the stasis described by Rüedi and Spoendlin (19), and Elonka and Applebaum (20).

Global analysis (6.63 μm) of the cochlea allowed for concomitant inspection of the round and oval windows. The otospongiotic bone at the FAF and its intense vascularization are clearly seen on the mesh reconstructions. The fibrotic tissue is seen on the 3D mesh images as a “red cloud,” but details are better visualized on the raw images. Further investigation of the round window (RW) raw images revealed fibrotic tissue connecting to the OSL, thickening of the CP, and accumulating CLs in the scala tympani region. The lithic formation is present under the CP and on the SG wall at the TP of the OSL. When these images are compared to synchrotron radiation phase contrast imaging (SR-PCI) full cochlea and local RW studies, the differences between a healthy and a diseased cochlea become clear (15, 21, 22). Chevance et al. and Bretlau et al., in 1970 and 1971, respectively (23–25), demonstrated increased levels of lysozymes on the otosclerotic focus, which suggests a local immunological response that leads to the tissue remodeling seen on this cochlea as fibrosis and CLs.

Reviewing the 4.43-μm raw dataset simultaneously with the 3D mesh image used to create the tonotopic map with the OSL as reference, revealed the presence of a CL on top of the 2 kHz area. The Carhart notch is a classical sign of otosclerosis seen in the audiogram as a notch at the 2 kHz frequency in bone conduction evaluation and suggests mechanical immobilization of the stapes footplate. Although no audiological evidence that this patient presented such a phenomenon was available, it is interesting that a calcified lesion was observed precisely on top of this position of the OSL and nowhere else. Histological studies have also failed to detect the presence of such calcifications inside the cochlea (1, 26). The authors believe that the processes involved in histological studies might explain why this is so.

Recent studies of the OSL demonstrated its movement in response to sound and the impact on bone conductive hearing (8, 9, 27, 28). The OSL moves through inertial vibration and resonates at 7 kHz, partially contributing to bone conduction sound (28). The images presented here support the hypothesis of the existence of an additional intracochlear factor (i.e., inflammatory responses) that might explain the clinical observation of poor outcomes in patients who had undergone stapedectomy and had the Carhart notch, reported by Kashio et al. (29). The significance of the Carhart notch in the diagnosis of stapes fixation was investigated by Kashio et al. (29), who compared its incidence in patients with other ossicular chain disorders (29). The authors reported a similar incidence of the notch in patients of all groups (stapes fixation, incudostapedial joint detachment, malleolus, or incus fixation) and concluded that the Carhart notch is not a good predictor of stapes fixation. This raises the question whether the notch seen on the audiogram is perhaps an initial consequence of the bone dysplasia and fibrotic formation already happening inside the cochlea. Future studies with a bigger sample size and audiological data should be performed to answer this question.

The modiolus was also rendered at this magnification and a reduction of the bony web that composes the human modiolus was noted on the otosclerotic cochlea (Figure 9), when compared to the healthy cochlea using microCT. Bony alterations have been described as a cause of SNHL in otosclerosis, as well as the presence of vascular shunts between the otosclerotic foci and the capillaries running to the modiolus (20). Although microCT cannot tell us whether there are any vascular alterations of the modiolus, the architectural difference becomes apparent when analyzing literature descriptions (14, 30) and our non-pathological dataset. Inspection of the cochlea turns at 2 μm revealed thickened cochlear walls, indicating the involvement of the SL and the stria vascularis as a result of the disease. The basal turn had an accumulation of fibrotic tissue at the RW and OW, with intense calcification of the CP. The midturn had a thickened CP, bridge, SL, and fibrosis over the OSL. Histologic studies have described atrophy, sclerotic, spongiotic and fibrous lesions, but there has so far been no description of calcifications (31–38).

The apex was completely filled with fibrotic tissue and calcifications, which is in line with classical disease findings such as low frequency hearing loss. Recent genetic studies (10) investigated a myriad of causes for otosclerosis including the role of the immune system, inflammation and oxidative stress, all leading to bone remodeling (39). The molecular mechanisms seem to involve the action of cytokines in the amplification of immunological responses. These chronic pro-inflammatory reactions can stimulate the accumulation of extracellular matrix proteins and have a deleterious impact on hearing performance. The buildup seen in this dataset matched the fibrotic formations and bone development described in histological studies (38–43). Comparing the radiological evidence seen in clinical CT with microCT leads to the conclusion that the disease in this patient is, in reality, far more advanced than what is recognizable in a normal clinical temporal bone CT scan (44). The clinically observed worsening of hearing (45) can sometimes be the only clue as to the ongoing intracochlear tissue remodeling.

When compared to reports in the literature, the OSL width did not differ (9). In contrast, our volumetric porosity study was very different from those previously described (9). However, no meaningful comparison can be made since Raufer et al. (9) used different imaging modalities, resolution, segmentation, and measurement methods. Volumetric reconstruction provides more information than 2D surfaces; therefore, the volumetric methodology seems to be the most suitable to calculate stiffness. The data presented here identified a slightly more porous vestibular plate (B: 53%; A: 57%) than tympanic plate (B: 51%; A: 53%), respectively in the basal turn (B) and the apex (A), while the midturn demonstrated the opposite behavior (VP, 54%; TP, 63%). This result contradicts what was described in the literature (9, 27), which usually reported the TP to be more porous than the VP, except on the midturn where the opposite was found.

## Conclusion

In this study, structural anatomical alterations of the otosclerotic cochlea were visualized in 3D for the first time, without the need for sample processing. The tonotopic map showed the presence of a cochleolith over the 2 kHz frequency. Interestingly, the volumetric 3D porosity measurement revealed results that contradict those of the known studies with 2D measurements. MicroCT suggested that even though the disease may not appear to be advanced in standard clinical CT scans, intense tissue remodeling is already ongoing inside the cochlea. That knowledge will have a considerable impact on further treatment of patients presenting with sensorineural hearing loss.

## Data availability statement

The raw data supporting the conclusions of this article will be made available by the authors, without undue reservation.

## Ethics statement

The studies involving human participants were reviewed and approved by KEK Bern, Switzerland, Project-ID 2018-00770. The patients/participants provided their written informed consent to participate in this study.

## Author contributions

GO'T: conceptualization, development of study design and methodology, validation, formal analysis of study data, data curation, writing original draft, and project administration. RZ and AP: software development, designing computer scans and supporting algorithms, validation, and performing the experiments. MB: formal analysis of study data, computing resources, and data curation. MC: validation of research output and supervision. FW: neuroradiology evaluation of the CT scans, formal analysis of study data, writing, reviewing, editing of the original draft, validation of research outputs, and supervision. All authors contributed to the article and approved the submitted version.

## Funding

Open access funding provided by Empa–Swiss Federal Laboratories for Materials Science and Technology.

## Conflict of interest

The authors declare that the research was conducted in the absence of any commercial or financial relationships that could be construed as a potential conflict of interest.

## Publisher's note

All claims expressed in this article are solely those of the authors and do not necessarily represent those of their affiliated organizations, or those of the publisher, the editors and the reviewers. Any product that may be evaluated in this article, or claim that may be made by its manufacturer, is not guaranteed or endorsed by the publisher.

## Abbreviations

3D: three-dimensional
CBCT: cone- beam computed tomography
CLs: cochleoliths
FAF: fissula ante fenestrum
IAC: internal auditory canal
ICV: inferior cochlear vein
OSL: osseous spiral lamina
OW: oval window
RW: round window
SD: standard deviation
SG: spiral ganglion
SL: spiral ligament
SNHL: sensorineural hearing loss
VP: vestibular plate.

## References

[1] Cureoglu S Schachern PA Ferlito A Rinaldo A Tsuprun V Paparella MM. Otosclerosis: etiopathogenesis and histopathology. Am J Otolaryngol. (2006) 27:334–40. 10.1016/j.amjoto.2005.11.001 16935179

[2] Guild S. R. Histologic otosclerosis. Ann Otol. (1944) 53:246–67.

[3] Schuknecht HF. Disorders of Bone. 2nd ed. Philadelphia, PA: Lea and Febiger (1993).

[4] Puac P Rodriguez A Lin HC Onofrj V Lin FC Hung SC. Cavitary plaques in otospongiosis: Ct findings and clinical implications. AJNR Am J Neuroradiol. (2018) 39:1135–9. 10.3174/ajnr.A5613 29622557 PMC6002895

[5] Fayad JN Makarem AO Linthicum FH Jr. Histopathologic assessment of fibrosis and new bone formation in implanted human temporal bones using 3d reconstruction. Otolaryngol Head Neck Surg. (2009) 141:247–52. 10.1016/j.otohns.2009.03.031 19643260 PMC2779735

[6] Danesh AA Shahnaz N Hall JW 3rd. The audiology of otosclerosis. Otolaryngol Clin North Am. (2018) 51:327–42. 10.1016/j.otc.2017.11.007 29397946

[7] Lee TC Aviv RI Chen JM Nedzelski JM Fox AJ Symons SP. Ct grading of otosclerosis. AJNR Am J Neuroradiol. (2009) 30:1435–9. 10.3174/ajnr.A1558 19321627 PMC7051554

[8] Raufer S Guinan JJ Jr Nakajima HH. Cochlear partition anatomy and motion in humans differ from the classic view of mammals. Proc Natl Acad Sci U S A. (2019) 116:13977–82. 10.1073/pnas.1900787116 31235601 PMC6628837

[9] Raufer SI Zosuls C Marino A Blanke G Bigio N O'Malley IJ. Anatomy of the human osseous spiral lamina and cochlear partition bridge: relevance for cochlear partition motion. J Assoc Res Otolaryngol. (2020) 21:171–82. 10.1007/s10162-020-00748-1 32166603 PMC7270316

[10] Babcock TA Liu XZ. Otosclerosis from genetic to molecular biology. Otolaryngol Clin North Am. (2018) 51:305–18. 10.1016/j.otc.2017.11.002 29502723

[11] Chen DA. Glasscock-shambaugh surgery of the Ear, 5th Ed. Otol Neurotol. (2003) 24:520. 10.1097/00129492-200305000-00028

[12] Li H Helpard L Ekeroot J Rohani SA Zhu N Rask-Andersen H. Three-dimensional tonotopic mapping of the human cochlea based on synchrotron radiation phase-contrast imaging. Sci Rep. (2021) 11:4437. 10.1038/s41598-021-83225-w 33627724 PMC7904830

[13] Greenwood DD. Critical bandwidth and the frequency coordinates of the basilar membrane. J Acoust Soc Am. (1961) 33:1344–56. 10.1121/1.1908437

[14] Iyer JS Zhu N Gasilov S Ladak HM Agrawal SK Stankovic KM. Visualizing the 3d cytoarchitecture of the human cochlea in an intact temporal bone using synchrotron radiation phase contrast imaging. Biomed Opt Express. (2018) 9:3757–67. 10.1364/BOE.9.003757 30338153 PMC6191620

[15] Lareida A Beckmann F Schrott-Fischer A Glueckert R Freysinger W Muller B. High-resolution X-ray tomography of the human inner ear: synchrotron radiation-based study of nerve fibre bundles, membranes and ganglion cells. J Microsc. (2009) 234:95–102. 10.1111/j.1365-2818.2009.03143.x 19335460

[16] Shim YJ Bae YJ An GS Lee K Kim Y Lee SY. Involvement of the internal auditory canal in subjects with cochlear otosclerosis: a less acknowledged third window that affects surgical outcome. Otol Neurotol. (2019) 40:e186–90. 10.1097/MAO.0000000000002144 30741893

[17] Hall IS Ogilvie RF. Otosclerosis in identical twins. J Laryngol Otol. (1954) 68:785–804. 10.1017/S0022215100050295 13212264

[18] Rüedi L. Otosclerotic lesion and cochlear degeneration. Arch Otolaryngol. (1969) 89:364–71. 10.1001/archotol.1969.00770020366023 5763932

[19] Rüedi L. Spoendlin, H. XL Pathogenesis of Sensorineural Deafness in Otosclerosis. Ann Otol Rhinol Laryngol. (1966) 75:525–52. 10.1177/000348946607500218 5912894

[20] Elonka DR Applebaum EL. Otosclerotic involvement of the cochlea: a histologic and audiologic study. Otolaryngol Head Neck Surg. (1981) 89:343–51. 10.1177/019459988108900239 6787539

[21] Atturo F Schart-Moren N Larsson S Rask-Andersen H Li H. The human cochlear aqueduct and accessory canals: a micro-Ct Analysis using a 3d reconstruction paradigm. Otol Neurotol. (2018) 39:e429–e35. 10.1097/MAO.0000000000001831 29794687

[22] Topperwien M Gradl R Keppeler D Vassholz M Meyer A Hessler R. Propagation-based phase-contrast x-ray tomography of cochlea using a compact synchrotron source. Sci Rep. (2018) 8:4922. 10.1038/s41598-018-23144-5 29563553 PMC5862924

[23] Bretlau P Causse J Jorgensen MB Chevance LG. Histiocytic activity in the otosclerotic bone. Arch Klin Exp Ohren Nasen Kehlkopfheilkd. (1971) 198:301–16. 10.1007/BF00316931 5569459

[24] Chevance LG Bretlau P Jorgensen MB Causse J. Otosclerosis. An electron microscopic and cytochemical study. Acta Otolaryngol Suppl. (1970) 272:1–44. 4322963

[25] Chevance LG Causse J Bretlau P Jorgensen MB Berges J. Hydrolytic activity of the perilymph in otosclerosis. A preliminary report. Acta Otolaryngol. (1972) 74:23–8. 10.3109/00016487209128418 4341581

[26] Vasama JP Linthicum FH Jr. Temporal bone histopathology case of the month cochlear otosclerosis. Am J Otolaryngol. (1998) 19:398–9. 9596194

[27] Kucuk B Abe K Ushiki T Inuyama Y Fukuda S Ishikawa K. Microstructures of the bony modiolus in the human cochlea: a scanning electron microscopic study. J Electron Microsc. (1991) 40:193–7. 1791403

[28] Naohito Hato SP Goode RL. Basilar membrane and osseous spiral lamina motion in human cadavers with air and bone conduction. Stimuli Hear Res. (2003) 181:131–43. 10.1016/S0378-5955(03)00183-7 12855371

[29] Kashio A Ito K Kakigi A Karino S Iwasaki S Sakamoto T. Carhart notch 2-Khz bone conduction threshold dip: a nondefinitive predictor of stapes fixation in conductive hearing loss with normal tympanic membrane. Arch Otolaryngol Head Neck Surg. (2011) 137:236–40. 10.1001/archoto.2011.14 21422306

[30] Elfarnawany M Alam SR Rohani SA Zhu N Agrawal SK Ladak HM. Micro-Ct versus synchrotron radiation phase contrast imaging of human cochlea. J Microsc. (2017) 265:349–57. 10.1111/jmi.12507 27935035

[31] Johnsson LG Hawkins JE Jr. Strial atrophy in clinical and experimental deafness. Laryngoscope. (1972) 82:1105–25. 10.1288/00005537-197207000-00002 5078636

[32] Allam AF. Pathology of the human spiral ligament. J Laryngol Otol. (1970) 84:765–79. 10.1017/S0022215100072534 5456805

[33] Wright JL Schuknecht HF. Atrophy of the spiral ligament. Arch Otolaryngol. (1972) 96:16–21. 10.1001/archotol.1972.00770090054005 5032052

[34] Holleman IL Jr Harrill JA. Cochlear otosclerosis report of a case, with postmortem histologic findings. Laryngoscope. (1967) 77:493–507. 10.1288/00005537-196704000-00004 6023610

[35] Kerr A Schuknecht HF. The spiral ganglion in profound deafness. Acta Otolaryngol. (1968) 65:586–98. 10.3109/00016486809119293 5706029

[36] Altmann F Kornfeld M Shea JJ. Inner ear changes in otosclerosis, histopathologic studies. Ann Otol Rhinol Laryngol. (1966) 75:5–32. 10.1177/000348946607500101 5929517

[37] Carhart R. Labyrinthine otosclerosis. Clinical manifestations of retrofenestral otosclerosis. Arch Otolaryngol. (1963) 78:477–508. 10.1001/archotol.78.4.91 14059346

[38] Linthicum FH. Histopathology of otosclerosis. Otolaryngol Clin North Am. (1993) 26:335–52. 10.1016/S0030-6665(20)30813-6 8341566

[39] Burghard A Lenarz T Kral A Paasche G. Insertion site and sealing technique affect residual hearing and tissue formation after cochlear implantation. Hear Res. (2014) 312:21–7. 10.1016/j.heares.2014.02.002 24566091

[40] Li PM Somdas MA Eddington DK Nadol JB Jr. Analysis of intracochlear new bone and fibrous tissue formation in human subjects with cochlear implants. Ann Otol Rhinol Laryngol. (2007) 116:731–8. 10.1177/000348940711601004 17987778

[41] Busby PA Plant KL Whitford LA. electrode impedance in adults and children using the nucleus 24 cochlear implant system. Cochlear Implants Int. (2002) 3:87–103. 10.1179/cim.2002.3.2.87 18792117

[42] Turner CW Gantz BJ Karsten S Fowler J Reiss LA. Impact of hair cell preservation in cochlear implantation: combined electric and acoustic hearing. Otol Neurotol. (2010) 31:1227–32. 10.1097/MAO.0b013e3181f24005 20802370 PMC2957190

[43] Lindsay JR Beal DD. Sensorineural deafness in otosclerosis, observations on histopathology. Ann Otol Rhinol Laryngol. (1966) 75:436–57. 10.1177/000348946607500213 5912894

[44] Rodriguez-Antuna J Fernandez-Armendariz P Diaz-Valino JL. Otosclerosis: the halo sign. Acta Otorrinolaringol Esp. (2016) 67:57. 10.1016/j.otoeng.2014.06.003 25308795

[45] Wiatr A Skladzien J Strek P Wiatr M. Carhart notch-a prognostic factor in surgery for otosclerosis. Ear Nose Throat J. (2021) 100:NP193–7. 10.1177/0145561319864571 31558062

